# Hypnosis as a Mechanism of Emotion Regulation and Self-Integration: An Integrative Review of Neural, Cognitive, and Experiential Pathways to Fundamental Peace

**DOI:** 10.3390/bs16030395

**Published:** 2026-03-09

**Authors:** Luis Miguel Gallardo, Saamdu Chetri

**Affiliations:** Yogananda School of Spirituality and Happiness, Shoolini University, Solan 173229, Himachal Pradesh, India; dr.saamdu.chetri@gmail.com

**Keywords:** hypnosis, emotion regulation, self-integration, default mode network, executive control, salience network, dissociation, neuroimaging, Fundamental Peace, trauma

## Abstract

Hypnosis has traditionally been conceptualized as a clinical technique for reducing physiological symptoms (e.g., pain, nausea) and psychological symptoms (e.g., anxiety, intrusive thoughts), yet emerging neuroscientific evidence suggests it operates through the fundamental mechanisms of emotional regulation and self-integration. This integrative review synthesizes research on clinical hypnosis from cognitive neuroscience, affective science, and clinical practice to examine how hypnotic phenomena modulate large-scale brain networks—particularly the default mode network (DMN), executive control network (ECN), and salience network (SaN)—to reorganize emotional experience and self-referential processing. We propose a formal mechanistic model in which hypnotic induction produces heightened experiential plasticity through coordinated network reconfiguration, enabling adaptive emotion regulation and reduced dissociative fragmentation. Central to this framework is the construct of Fundamental Peace (FP), operationalized as a dynamic neuro-experiential state characterized by: (1) flexible attentional control without effortful suppression; (2) emotional coherence across self-states; (3) reduced self-referential rigidity; (4) compassionate self-awareness. Unlike equanimity (affective neutrality) or well-being (positive evaluation), Fundamental Peace represents integrated regulatory capacity under changing conditions. Key findings from neuroimaging studies demonstrate that hypnotic states consistently reduce DMN activity, enhance ECN-SaN coupling, and modulate connectivity patterns associated with self-referential processing. Meta-analytic evidence from 85 controlled experimental trials shows robust pain reduction effects, while clinical studies document improvements in trauma-related dissociation and emotional dysregulation. We critically evaluate this framework against alternative theories (dissociated control, cold control, predictive processing, social-cognitive models), specify testable predictions, and assess evidence quality across neuroimaging and clinical domains. Implications for trauma treatment, clinical implementation, and future research integrating causal inference methods are discussed, alongside ethical and cultural considerations.

## 1. Introduction

### 1.1. Historical Context and Contemporary Understanding

Hypnosis represents one of the most intriguing phenomena in psychological science, offering a unique window into the malleability of consciousness, emotion, and self-experience. Recent integrative models have redefined hypnosis not merely as a clinical technique but as a complex psychobiological phenomenon involving coordinated changes across cognitive, affective, and neurophysiological systems (Geagea et al., 2023). While historically viewed primarily as a clinical tool for symptom management—particularly pain relief, anxiety reduction, and habit modification—contemporary neuroscience research reveals that hypnotic states engage fundamental mechanisms of brain network organization, attentional control, and self-referential processing (Oakley & Halligan, 2013).

The history of clinical hypnosis spans more than two centuries, beginning with Franz Anton Mesmer’s work on “animal magnetism” in the late 18th century (Gauld, 1992). The term “hypnosis” was coined by James Braid in 1843, derived from the Greek word “hypnos” (sleep), though Braid later recognized that hypnosis was not actually a sleep state (Braid, 1843). The late 19th and early 20th centuries saw significant contributions from Jean-Martin Charcot, who studied hypnosis in relation to hysteria at the Salpêtrière Hospital in Paris, and Pierre Janet, who developed theories of dissociation that remain influential today (Janet, 1889; Ellenberger, 1970).

Two figures stand as particularly influential icons in the modern development of clinical hypnosis: Milton H. Erickson and Herbert Spiegel. Milton Erickson (1901–1980) revolutionized the field through his development of indirect, permissive, and naturalistic approaches to hypnotic induction and suggestion (Erickson, 1980; Haley, 1973). Unlike traditional authoritarian methods, Erickson emphasized utilizing the patient’s own resources, experiences, and language patterns to facilitate therapeutic change. His work demonstrated that hypnosis could be tailored to individual differences in responsiveness and that therapeutic suggestions could be embedded within conversational narratives rather than delivered as direct commands (Erickson & Rossi, 1979). Erickson’s contributions extended beyond technique to encompass a philosophical approach emphasizing patient autonomy, creative problem-solving, and the mobilization of unconscious resources for healing. His legacy continues through Ericksonian hypnotherapy training programs worldwide and has influenced contemporary approaches to brief therapy, solution-focused therapy, and narrative therapy (Zeig, 1985).

Herbert Spiegel (1914–2009), along with his son David Spiegel, made equally significant contributions through systematic research on hypnotizability and the development of standardized assessment methods. Herbert Spiegel developed the Hypnotic Induction Profile (HIP), a brief clinical assessment tool that evaluates hypnotic responsiveness through eye-roll induction and standardized suggestions (H. Spiegel & Spiegel, 2004). The HIP represented a major advance in making hypnotizability assessment practical for clinical settings, requiring only 5–10 min to administer compared to the hour-long Stanford Hypnotic Susceptibility Scales. Spiegel’s work emphasized the biological basis of hypnotic capacity, proposing that hypnotizability represents a stable trait with neurobiological correlates (D. Spiegel, 1974). His research demonstrated that hypnotic responsiveness could predict treatment outcomes and that understanding individual differences in hypnotizability was essential for effective clinical application. The Spiegels’ textbook “Trance and Treatment” (first published in 1978, revised in 2004) remains a foundational clinical resource, integrating assessment, theory, and practical therapeutic applications (H. Spiegel & Spiegel, 2004).

The past three decades have witnessed a surge of neuroscientific research examining the neural correlates of hypnotic states and responses to hypnotic suggestions (Oakley & Halligan, 2013; Raz & Lifshitz, 2016). Neuroimaging studies using functional magnetic resonance imaging (fMRI), positron emission tomography (PET), and electroencephalography (EEG) have revealed consistent patterns of brain activity changes during hypnosis, particularly involving the default mode network, executive control network, and salience network (Demertzi et al., 2011; McGeown et al., 2009; Rainville et al., 1999). These findings have shifted theoretical understanding from viewing hypnosis as a purely psychological phenomenon to recognizing it as involving measurable alterations in large-scale brain network dynamics.

### 1.2. Clinical Applications and Evidence Base

The clinical applications of hypnosis have expanded considerably beyond its historical association with pain management. A comprehensive systematic review and meta-analysis of 85 controlled experimental trials by Thompson et al. (2019) demonstrated robust effects of hypnosis for pain relief, with effect sizes varying by pain type and hypnotic suggestibility. This meta-analysis, published in Neuroscience & Biobehavioral Reviews, analyzed data from over 3600 participants and found that hypnotic analgesia produced significant pain reduction across diverse experimental pain paradigms, including thermal, pressure, ischemic, and cold pressor pain. Importantly, Thompson et al. (2019) found that effects were moderated by individual differences in hypnotic suggestibility, with highly suggestible individuals showing substantially larger pain reductions than low suggestible individuals. The review also identified specific neural mechanisms underlying hypnotic analgesia, including modulation of anterior cingulate cortex activity and altered connectivity between sensory and affective pain processing regions.

Beyond pain management, clinical hypnosis has demonstrated efficacy for pediatric populations across various medical procedures. Geagea et al. (2023) reviewed evidence for hypnosis in pediatric settings, highlighting its utility for reducing procedural anxiety, managing chronic pain conditions, and facilitating recovery from surgical procedures. Their integrative model emphasizes that hypnosis in children may operate through somewhat different mechanisms than in adults, with greater reliance on imaginative engagement and less emphasis on formal induction procedures. Pediatric applications have shown particular promise for conditions such as functional abdominal pain, needle-related anxiety, and burn wound care (Birnie et al., 2014; Liossi & Hatira, 2003).

Contemporary research has also documented applications in trauma treatment, anxiety disorders, depression, habit disorders (smoking cessation, weight management), and psychosomatic conditions (Flammer & Alladin, 2007; Lynn et al., 2015). The mechanisms underlying these diverse clinical effects, however, remain incompletely understood, particularly regarding how hypnotic interventions produce lasting changes in emotional regulation and self-experience.

### 1.3. Rationale and Knowledge Gaps

Despite the growing evidence base for clinical hypnosis and increasing understanding of its neural correlates, several critical knowledge gaps remain. First, while numerous studies have documented brain activity changes during hypnosis, the field lacks integrative theoretical models that coherently link these neural changes to specific psychological mechanisms and clinical outcomes. Most existing theories focus on single dimensions—such as attentional control (Egner & Raz, 2007), dissociation (Woody & Bowers, 1994), or social-cognitive factors (Lynn et al., 2015)—without adequately integrating across levels of analysis.

Second, the relationship between hypnotic interventions and long-term changes in emotional regulation capacity remains poorly characterized. While acute effects on pain, anxiety, and other symptoms are well-documented, the mechanisms by which hypnosis might produce enduring changes in self-regulatory capacity and emotional integration are not well understood. This gap is particularly significant for trauma treatment applications, where the goal is not merely symptom reduction but fundamental reorganization of fragmented self-states and maladaptive emotion regulation patterns.

Third, existing research has not adequately addressed how hypnotic interventions might facilitate states of integrated well-being that extend beyond symptom relief. Most clinical outcome studies focus on reduction in negative symptoms rather than enhancement of positive psychological capacities such as emotional coherence, self-compassion, and adaptive flexibility. This limitation reflects a broader gap in the field’s conceptual frameworks, which have historically emphasized pathology reduction over wellness promotion.

Fourth, the field lacks clear operational definitions and measurement approaches for constructs related to integrated self-experience and emotional coherence. While concepts such as “ego integration,” “self-coherence,” and “psychological well-being” appear frequently in clinical literature, they are rarely operationalized in ways that permit rigorous empirical investigation or clear linkage to neural mechanisms.

This integrative review addresses these gaps by proposing a comprehensive mechanistic framework that links hypnotic modulation of large-scale brain networks to specific changes in emotion regulation capacity and self-integration. Central to this framework is the construct of Fundamental Peace (FP), which we operationalize as a measurable neuro-experiential state characterized by flexible attentional control, emotional coherence across self-states, reduced self-referential rigidity, and compassionate self-awareness. By integrating evidence from cognitive neuroscience, affective science, and clinical hypnosis research, we aim to provide a theoretically grounded and empirically testable model for understanding how hypnotic interventions can facilitate lasting changes in emotional regulation and self-experience.

## 2. Theoretical Framework: Large-Scale Brain Networks and Hypnotic States

### 2.1. The Triple Network Model

Understanding the neural mechanisms of hypnosis requires consideration of how hypnotic states modulate large-scale brain networks that organize cognitive and emotional processing. The triple network model, proposed by Menon (2011), provides a useful framework for conceptualizing these dynamics. This model identifies three core networks that play central roles in cognition, emotion, and self-referential processing: the default mode network (DMN), the executive control network (ECN), and the salience network (SaN).

The default mode network comprises the medial prefrontal cortex, posterior cingulate cortex, precuneus, and angular gyrus. This network is typically active during rest and self-referential thinking, supporting autobiographical memory retrieval, future planning, theory of mind, and the construction of a continuous sense of self across time (Raichle et al., 2001; Buckner et al., 2008). Hyperactivity or dysregulated connectivity within the DMN has been associated with rumination, self-focused worry, and various forms of psychopathology, including depression and anxiety disorders (Hamilton et al., 2015). In the context of hypnosis, modulation of DMN activity may be particularly relevant for altering self-referential processing and reducing maladaptive patterns of self-focused attention.

The executive control network includes the dorsolateral prefrontal cortex and posterior parietal cortex. This network supports goal-directed attention, working memory, cognitive flexibility, and top-down regulation of cognition and emotion (Miller & Cohen, 2001). The ECN is critical for implementing cognitive control strategies, including attentional focus, response inhibition, and the regulation of emotional responses through reappraisal and other deliberate strategies. During hypnosis, enhanced ECN engagement may facilitate focused attention on suggestions and enable the implementation of suggested alterations in perception, cognition, or behavior.

The salience network comprises the anterior cingulate cortex and anterior insula. This network functions to detect and orient attention toward salient, whether external (environmental threats or opportunities) or internal (bodily sensations, emotional states). The SaN plays a crucial role in switching between the DMN and ECN, determining which network should be dominant at any given moment based on the salience of current stimuli or goals (Menon & Uddin, 2010). In hypnotic contexts, the SaN may be particularly important for directing attention toward hypnotic suggestions and away from competing stimuli or self-referential thoughts.

**Figure 1:**
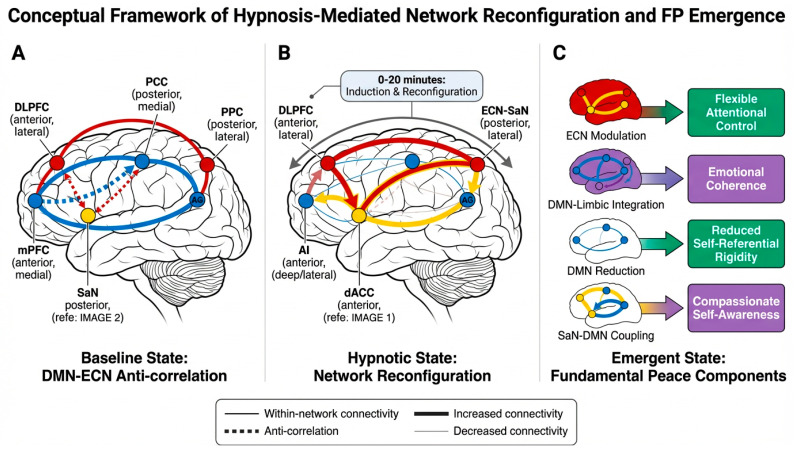
Conceptual Framework of Hypnosis-Mediated Network Reconfiguration and FP Emergence. **Three-panel structure:** (**A**) **(Enhanced):** Baseline network organization showing typical DMN-ECN anti-correlation; (**B**): Network reconfiguration during hypnotic induction (DMN reduction, ECN-SaN coupling, altered DMN-ECN connectivity); (**C**): Emergence of FP components from network changes.

We propose that hypnotic emotion regulation emerges from a three-stage process of coordinated network reconfiguration (see Figure 1 description below):

### 2.2. Network Dynamics During Hypnosis

Neuroimaging research has consistently demonstrated that hypnotic states involve coordinated changes across these three networks, though the specific patterns vary depending on the type of hypnotic induction, the content of suggestions, and individual differences in hypnotic suggestibility. Several key findings have emerged from this literature:

First, hypnotic induction typically produces decreased activity in the default mode network, particularly in the posterior cingulate cortex and medial prefrontal cortex (McGeown et al., 2009; Demertzi et al., 2011). This DMN reduction has been interpreted as reflecting decreased self-referential processing and reduced mind-wandering, consistent with the focused, absorbed quality of hypnotic experience. McGeown et al. (2009) found that the magnitude of DMN deactivation during hypnotic induction correlated with behavioral measures of hypnotic depth, suggesting that DMN modulation is functionally significant for the hypnotic state itself rather than merely an epiphenomenon.

Second, hypnotic states often involve increased functional connectivity between the executive control network and salience network, suggesting enhanced coordination between attentional control systems and salience detection mechanisms (Demertzi et al., 2011). This enhanced ECN-SaN coupling may enable the characteristic hypnotic capacity for focused attention combined with heightened responsiveness to suggestions. Importantly, this pattern differs from both normal waking states (where ECN and SaN show more independent operation) and sleep states (where both networks show reduced activity).

Third, specific hypnotic suggestions produce targeted modulation of brain regions relevant to the suggested experience. For example, suggestions for pain reduction modulate activity in the anterior cingulate cortex and insula—regions involved in the affective dimension of pain—while leaving sensory pain processing regions relatively intact (Rainville et al., 1997; Vanhaudenhuyse et al., 2009). Similarly, suggestions for visual hallucinations modulate activity in visual cortex, while suggestions for motor paralysis affect motor planning regions (Oakley & Halligan, 2013). This specificity suggests that hypnotic suggestions do not produce global, non-specific changes in brain function but rather targeted alterations in specific neural systems relevant to the suggested experience.

It is important to note that these network dynamics show considerable individual variability, with highly hypnotizable individuals typically showing more pronounced network changes than low hypnotizable individuals (Hoeft et al., 2012). Additionally, while the evidence for DMN reduction during hypnosis is relatively consistent, the specific patterns of ECN and SaN modulation may vary depending on the particular hypnotic procedures used and the cognitive demands of specific suggestions. Some studies have found increased ECN activity during hypnosis, while others have found decreased activity, likely reflecting differences in whether participants are actively engaged in effortful cognitive tasks or are in a more passive, receptive state (Oakley & Halligan, 2013). Further research using standardized protocols and larger samples is needed to fully characterize the range of network configurations associated with different hypnotic phenomena.

### 2.3. Absorption, Dissociation, and Network Connectivity

Individual differences in hypnotic responsiveness have been linked to trait characteristics such as absorption—the capacity for deep imaginative involvement and focused attention (Tellegen & Atkinson, 1974)—and dissociative capacity—the ability to compartmentalize aspects of experience or consciousness (Butler et al., 1996). These traits may relate to baseline differences in brain network organization and connectivity.

Research suggests that individuals high in absorption show distinctive patterns of resting-state functional connectivity, particularly involving the default mode network and attentional networks (Demertzi et al., 2011). Some evidence indicates that high absorbers may have greater baseline connectivity between DMN and ECN regions, potentially facilitating the rapid network reconfiguration that occurs during hypnotic induction. However, the relationship between absorption, dissociation, and network connectivity remains an area of active investigation, with some inconsistencies across studies.

The relationship between dissociative capacity and hypnotic responsiveness is complex and somewhat controversial. While clinical dissociation (as seen in dissociative disorders) and hypnotic responsiveness both involve alterations in consciousness and self-experience, they appear to be distinct phenomena with different neural correlates and functional consequences (Butler et al., 1996). Pathological dissociation typically involves fragmentation of self-experience and impaired integration across self-states, whereas hypnotic responsiveness involves flexible, controlled alterations in consciousness that do not necessarily involve fragmentation. Some researchers have proposed that hypnosis may actually facilitate integration of dissociated self-states in trauma survivors, rather than producing further dissociation (D. Spiegel, 1990; van der Hart et al., 2006).

It should be acknowledged that the evidence linking absorption and dissociation to specific patterns of brain network connectivity is still emerging, and causal relationships remain unclear. While correlational studies have identified associations between these traits and connectivity patterns, experimental studies manipulating network connectivity to examine effects on hypnotic responsiveness are lacking. Additionally, most existing studies have used relatively small samples and have not always adequately controlled for potential confounds such as general cognitive ability, attentional capacity, or personality factors. Future research employing larger samples, longitudinal designs, and more sophisticated network analysis methods will be needed to fully elucidate these relationships.

## 3. Hypnotic Suggestibility and Assessment Methods

### 3.1. Individual Differences in Hypnotic Responsiveness

Hypnotic suggestibility, also termed hypnotic responsiveness or hypnotizability, refers to the degree to which an individual responds to hypnotic suggestions following a standardized induction procedure. This trait shows substantial individual variability, with approximately 10–15% of the population classified as highly hypnotizable, 10–15% as low hypnotizable, and the majority falling in the moderate range (Hilgard, 1965). Hypnotic suggestibility appears to be a relatively stable trait across the lifespan, though some evidence suggests modest increases during childhood and adolescence followed by gradual decline in older adulthood (Morgan & Hilgard, 1973).

Research has consistently demonstrated that hypnotic suggestibility predicts response to hypnotic interventions across diverse clinical applications. In pain management, for example, highly hypnotizable individuals typically show substantially greater pain reduction in response to hypnotic analgesia suggestions than low hypnotizable individuals (Thompson et al., 2019). Similarly, in smoking cessation, anxiety treatment, and other applications, treatment outcomes are often moderated by baseline hypnotizability (Lynn et al., 2015). These findings underscore the importance of assessing hypnotic responsiveness when considering hypnosis as a clinical intervention.

### 3.2. Standardized Assessment Instruments

Several standardized scales have been developed to assess hypnotic suggestibility in research and clinical contexts. The most widely used research instruments are the Stanford Hypnotic Susceptibility Scales (SHSS), developed by Weitzenhoffer and Hilgard (1959, 1962). The SHSS includes three forms (A, B, and C) that involve a standardized induction followed by a series of suggestions of increasing difficulty, ranging from simple motor suggestions (e.g., arm lowering) to more complex cognitive suggestions (e.g., age regression, hallucinations). The SHSS provides a quantitative score reflecting the number of suggestions to which the individual responds, with scores ranging from 0 to 12. While the SHSS is considered the gold standard for research purposes, its administration requires approximately one hour, limiting its practicality for routine clinical use.

The Harvard Group Scale of Hypnotic Susceptibility (HGSHS) represents an adaptation of the Stanford scales for group administration (Shor & Orne, 1962). The HGSHS can be administered to multiple individuals simultaneously, making it more efficient for screening large samples. However, group administration may reduce sensitivity to individual differences and does not permit the individualized rapport-building that characterizes clinical hypnosis practice.

### 3.3. The Hypnotic Induction Profile (HIP)

A particularly significant contribution to clinical assessment of hypnotizability is the Hypnotic Induction Profile (HIP), developed by Herbert Spiegel and refined in collaboration with his son David Spiegel (H. Spiegel & Spiegel, 2004). The HIP was designed specifically for clinical settings, requiring only 5–10 min to administer while providing reliable assessment of hypnotic capacity. The HIP has become one of the most widely used clinical assessment tools for hypnotizability, particularly in medical and psychiatric settings where time constraints preclude use of longer research instruments.

The HIP procedure begins with the eye-roll sign, in which the patient is asked to look upward while slowly closing their eyelids. The degree of sclera visible during this maneuver is scored on a 0–4 scale, with higher scores indicating greater eye-roll capacity. Spiegel proposed that the eye-roll sign reflects neurological factors related to hypnotic capacity, though this interpretation remains somewhat controversial (D. Spiegel, 1974). Following the eye-roll, the clinician administers a brief induction involving arm levitation, followed by several test suggestions including arm levitation, dissociation between hands, and post-hypnotic suggestion. The patient’s responses are scored to yield an overall hypnotizability profile classified as intact (highly hypnotizable), mid-range, or decremented (low hypnotizability).

The HIP offers several advantages for clinical practice. First, its brevity makes it practical for routine clinical assessment. Second, the procedure itself serves as a brief hypnotic experience, allowing patients to directly experience hypnosis while being assessed. Third, the HIP provides not only a quantitative score but also qualitative information about the patient’s hypnotic style and potential clinical applications. H. Spiegel and Spiegel (2004) describe distinct hypnotic profiles associated with different personality styles and clinical presentations, providing guidance for tailoring hypnotic interventions to individual characteristics.

Research on the HIP has demonstrated moderate correlations with longer standardized scales such as the Stanford scales, with correlation coefficients typically in the 0.4–0.6 range (D. Spiegel et al., 1976). While these correlations indicate that the HIP assesses related constructs, the moderate magnitude suggests that the HIP may capture somewhat different aspects of hypnotic responsiveness than the Stanford scales. Some researchers have suggested that the HIP may be particularly sensitive to dissociative capacity and imaginative involvement, while the Stanford scales may place greater emphasis on behavioral compliance with suggestions (Frischholz et al., 1987). Despite some psychometric limitations, the HIP remains widely used in clinical practice due to its practicality and clinical utility.

### 3.4. Clinical Implications of Assessment

Understanding individual differences in hypnotic responsiveness has important implications for clinical practice. For highly hypnotizable individuals, hypnotic interventions can be a powerful and efficient treatment modality, often producing rapid symptom relief with relatively brief interventions. For individuals with moderate hypnotizability, hypnotic interventions may still be beneficial but may require more extended practice or combination with other therapeutic approaches. For low hypnotizable individuals, traditional hypnotic approaches may be less effective, though some evidence suggests that responsiveness can be enhanced through training, practice, and modification of induction procedures to match individual preferences and cognitive styles (Gorassini & Spanos, 1986).

It is important to note that low hypnotizability does not preclude benefit from hypnotic interventions. Many therapeutic elements of hypnosis—such as relaxation, focused attention, positive imagery, and therapeutic suggestions—can be beneficial even for individuals who do not experience classic hypnotic phenomena such as involuntariness or profound alterations in consciousness. Additionally, some clinicians argue that formal assessment of hypnotizability may be unnecessary in clinical practice, as therapeutic benefits can be achieved through flexible, individualized approaches that adapt to each patient’s responsiveness (Lynn et al., 2015). Nonetheless, assessment can provide valuable information for treatment planning and can help set realistic expectations for both clinician and patient.

## 4. Fundamental Peace: Conceptualization and Operationalization

### 4.1. Defining Fundamental Peace

Central to our integrative framework is the construct of Fundamental Peace (FP), which we propose as a measurable neuro-experiential state that can emerge through hypnotic modulation of emotion regulation and self-integration processes. Fundamental Peace is operationally defined as a dynamic state characterized by four core components:

**(1) Flexible attentional control without effortful suppression:** The capacity to direct and sustain attention according to current goals and values while remaining open to relevant information, without requiring constant effortful inhibition of distracting stimuli or thoughts. This differs from rigid attentional focus (which requires sustained effort and produces fatigue) and from uncontrolled mind-wandering (which lacks goal-directedness). FP involves a quality of effortless attention that has been described in contemplative traditions as “relaxed alertness” or “choiceless awareness.”

**(2) Emotional coherence across self-states:** The experience of continuity and integration across different emotional states and self-aspects, without fragmentation or dissociative compartmentalization. This includes the capacity to access and integrate emotional experiences from different life periods and contexts, to recognize connections between current emotional responses and past experiences, and to maintain a sense of self-continuity despite changes in emotional state. Emotional coherence does not imply emotional uniformity or the absence of emotional complexity, but rather the integration of diverse emotional experiences within a coherent self-narrative.

**(3) Reduced self-referential rigidity:** Decreased tendency toward rigid, repetitive self-referential thinking patterns such as rumination, self-criticism, or defensive self-enhancement. This component involves flexibility in self-representation, the capacity to view oneself from multiple perspectives, and reduced attachment to fixed self-concepts. Importantly, reduced self-referential rigidity does not imply absence of self-awareness or self-reflection, but rather a more flexible, adaptive quality of self-related cognition.

**(4) Compassionate self-awareness:** The capacity to observe one’s own experiences, thoughts, and emotions with an attitude of kindness, acceptance, and non-judgment. This includes self-compassion in the face of personal failures or limitations, the ability to acknowledge difficult emotions without being overwhelmed by them, and a balanced perspective that recognizes both strengths and weaknesses without excessive self-criticism or self-aggrandizement.

See Table 1 for Components of Fundamental Peace, Opearational Definition, Their Neural Correlates, and measurement Approaches. 

### 4.2. Distinguishing Fundamental Peace from Related Constructs

See how Table 2 systematically compares Fundamental Peace with related constructs:

Fundamental Peace is conceptually distinct from several related constructs in the psychological and contemplative literature, though it shares features with each.

**Equanimity** refers to a state of emotional balance and non-reactivity, often described as “even-mindedness” in Buddhist psychology (Desbordes et al., 2015). While equanimity emphasizes affective neutrality and non-reactivity to pleasant and unpleasant experiences, Fundamental Peace encompasses a broader range of capacities including attentional flexibility, self-integration, and compassionate awareness. An individual might experience equanimity (emotional balance) without necessarily having emotional coherence across self-states or compassionate self-awareness.

**Psychological well-being**, as conceptualized by Ryff (2014) and others, includes dimensions such as self-acceptance, positive relations with others, autonomy, environmental mastery, purpose in life, and personal growth. Eudaimonic well-being, a related concept, emphasizes meaning, purpose, and self-realization rather than hedonic pleasure or positive affect (Ryff, 2014; Waterman, 1993). Eudaimonic well-being is derived from Aristotle’s concept of “eudaimonia,” often translated as “flourishing” or “the good life,” and refers to living in accordance with one’s deeply held values and realizing one’s true potential. While FP shares some features with psychological and eudaimonic well-being—particularly self-acceptance and personal growth—FP is more specifically focused on the integration of emotion regulation capacities and the quality of self-referential processing. Well-being is a broader evaluative construct that includes life satisfaction and positive functioning across multiple domains, whereas FP is a more circumscribed neuro-experiential state.

**Flow** describes a state of complete absorption in an activity, characterized by focused attention, loss of self-consciousness, and intrinsic enjoyment (Csikszentmihalyi, 1990). While flow shares with FP the quality of effortless attention and reduced self-referential thinking, flow is specifically tied to engagement in challenging activities that match one’s skill level. FP, in contrast, is not necessarily activity-dependent and includes components (emotional coherence, compassionate self-awareness) that are not central to flow experiences.

**Mindfulness** involves present-moment awareness with an attitude of acceptance and non-judgment (Kabat-Zinn, 1990). FP shares with mindfulness the emphasis on non-judgmental awareness and present-moment focus, but FP additionally emphasizes emotional coherence across time (not just present-moment awareness) and includes specific attentional capacities (flexible control without effortful suppression) that are not always emphasized in mindfulness definitions.

### 4.3. Measurement Approaches for FP

Operationalizing Fundamental Peace for empirical research requires multi-method assessment approaches that capture both subjective experiential qualities and objective neural and behavioral markers. We propose a three-level measurement framework:

#### 4.3.1. Level 1: Self-Report Measures

Self-report instruments can assess the subjective experiential components of FP. While no existing validated scale directly measures FP as we have defined it, several existing instruments capture related dimensions:

- The Five Facet Mindfulness Questionnaire (FFMQ; Baer et al., 2006) assesses non-reactivity, non-judgment, and present-moment awareness.
- The Self-Compassion Scale (SCS; Neff, 2003) measures compassionate self-awareness.
- The Experiences Questionnaire (EQ; Fresco et al., 2007) assesses decentering and reduced self-referential rigidity.
- The Absorption Scale (Tellegen & Atkinson, 1974) captures aspects of focused attention and experiential involvement.

A comprehensive FP assessment would ideally integrate items from these instruments or develop a new scale specifically designed to capture the four core components of FP. Such a scale would need to demonstrate adequate psychometric properties including internal consistency, test–retest reliability, and discriminant validity from related constructs such as general well-being, trait mindfulness, and emotional stability.

#### 4.3.2. Level 2: Behavioral and Cognitive Measures

Objective behavioral tasks can assess specific capacities associated with FP:

- Attentional control tasks (e.g., sustained attention to response task, attention network test) can measure flexible attentional control.
- Emotion regulation tasks (e.g., reappraisal paradigms, emotion interference tasks) can assess regulatory capacity.
- Self-referential processing tasks (e.g., self-reference effect paradigms, rumination induction tasks) can measure self-referential flexibility.
- Narrative coherence coding of autobiographical memory narratives can assess emotional coherence across self-states.

#### 4.3.3. Level 3: Neural Markers

Neuroimaging and neurophysiological measures can provide objective markers of the neural states associated with FP:

- Resting-state fMRI measures of DMN activity and connectivity can index self-referential processing patterns.
- Task-based fMRI during emotion regulation can assess ECN-SaN coupling and regulatory capacity.
- EEG measures of frontal alpha asymmetry and frontal midline theta can index attentional and regulatory states.
- Heart rate variability (HRV) can provide a peripheral marker of autonomic regulation associated with emotional flexibility.

Importantly, FP should be conceptualized as a state that can vary in intensity and duration, rather than a fixed trait. Individuals may experience FP states of varying depth and duration, and the capacity to access FP states may increase with practice through hypnotic training, meditation, or psychotherapy. Longitudinal assessment approaches that track changes in FP over time and in response to interventions will be essential for validating the construct and understanding its development and maintenance.

## 5. Mechanistic Model: From Network Modulation to Fundamental Peace

### 5.1. Overview of the Proposed Model

Our integrative model proposes that hypnotic induction and suggestion produce Fundamental Peace through a cascade of coordinated changes across neural, cognitive, and experiential levels. The model specifies explicit mechanistic pathways linking hypnotic procedures to FP emergence, organized into three sequential but overlapping phases:

#### 5.1.1. Phase 1: Network Reconfiguration

Hypnotic induction produces coordinated changes in large-scale brain network activity and connectivity, specifically:

- Reduction in default mode network activity, particularly in posterior cingulate cortex and medial prefrontal cortex.
- Enhanced coupling between executive control network and salience network.
- Modulation of connectivity between the DMN and ECN, shifting from typical anti-correlated patterns toward more flexible, context-dependent configurations.

#### 5.1.2. Phase 2: Cognitive–Affective Reorganization

Network reconfiguration enables specific changes in cognitive and emotional processing:

- Reduced self-referential rumination and rigid self-concept activation (resulting from DMN reduction).
- Enhanced attentional control and responsiveness to suggestions (resulting from ECN-SaN coupling).
- Increased emotional flexibility and reduced defensive processing (resulting from altered DMN-ECN connectivity).
- Facilitated access to and integration of dissociated or compartmentalized emotional experiences.

#### 5.1.3. Phase 3: Experiential Integration

Cognitive–affective reorganization produces the subjective experiential qualities of Fundamental Peace:

- Effortless attentional focus emerges from enhanced ECN-SaN coordination without requiring sustained DMN suppression.
- Emotional coherence emerges from reduced dissociative compartmentalization and enhanced integration across self-states.
- Self-referential flexibility emerges from reduced DMN rigidity and enhanced capacity for perspective-taking.
- Compassionate self-awareness emerges from reduced defensive processing and enhanced access to self-compassion resources.

### 5.2. Detailed Mechanistic Pathways

#### 5.2.1. Pathway 1: DMN Reduction → Reduced Self-Referential Rigidity

The reduction in default mode network activity during hypnotic induction directly impacts self-referential processing. The DMN, particularly the medial prefrontal cortex and posterior cingulate cortex, is consistently activated during self-referential thinking, autobiographical memory retrieval, and mental time travel (Buckner et al., 2008). Hyperactivity in these regions has been associated with rumination, self-focused worry, and rigid self-concepts in depression and anxiety disorders (Hamilton et al., 2015).

During hypnotic induction, focused attention on the hypnotist’s voice or on specific imagery reduces DMN activity, temporarily interrupting habitual self-referential thought patterns. This interruption creates a window of opportunity for reorganizing self-related cognition. Hypnotic suggestions can then be used to introduce more flexible, adaptive self-representations or to facilitate access to self-aspects that are typically inhibited or dissociated. For example, suggestions for self-compassion or self-acceptance may be more readily integrated when the usual defensive self-referential processing is temporarily reduced.

Importantly, the goal is not permanent suppression of DMN activity—which would be neither possible nor desirable—but rather temporary modulation that allows for reorganization of self-referential processing patterns. With repeated hypnotic practice, individuals may develop greater flexibility in DMN engagement, learning to shift between focused, task-oriented states (with reduced DMN activity) and reflective, self-referential states (with appropriate DMN engagement) according to current needs and goals.

#### 5.2.2. Pathway 2: ECN-SaN Coupling → Flexible Attentional Control

Enhanced functional connectivity between the executive control network and salience network during hypnosis facilitates a distinctive quality of attention characterized by both focus and flexibility. The ECN supports goal-directed attention and cognitive control, while the SaN detects salient stimuli and signals the need for attentional shifts (Menon & Uddin, 2010). In typical waking consciousness, these networks often operate somewhat independently, with the SaN triggering attentional shifts that may interrupt ECN-mediated goal pursuit.

During hypnosis, enhanced ECN-SaN coupling may enable a more integrated attentional state in which salience detection and attentional control are coordinated rather than competing. This coordination allows for sustained focus on hypnotic suggestions while remaining responsive to relevant changes in internal or external context. The subjective experience is one of effortless attention—focused yet flexible, sustained yet not rigid.

This attentional quality differs from both the effortful concentration required in many cognitive tasks (which produces mental fatigue and requires active suppression of distractors) and the unfocused mind-wandering of typical rest states (which lacks goal-directedness). The effortless attention of hypnotic states may represent an optimal configuration for learning and reorganization, as it combines the benefits of focused attention (enhanced encoding, reduced interference) with the benefits of relaxed awareness (reduced defensive processing, enhanced creativity).

#### 5.2.3. Pathway 3: Altered DMN-ECN Connectivity → Emotional Coherence

The relationship between the default mode network and executive control network is typically characterized by anti-correlation: when one network is active, the other tends to be suppressed (Fox et al., 2005). This anti-correlation is thought to reflect a fundamental distinction between internally focused, self-referential processing (DMN) and externally focused, goal-directed processing (ECN).

However, this strict anti-correlation may contribute to dissociative fragmentation in trauma survivors and others with emotion regulation difficulties. When self-referential processing (DMN) and executive control (ECN) operate in opposition, emotional experiences may become compartmentalized, with limited integration between emotional memories (DMN-mediated) and current goal-directed behavior (ECN-mediated). This compartmentalization can manifest as dissociative symptoms, emotional numbing, or difficulty integrating traumatic experiences into coherent autobiographical narratives.

Hypnotic states may temporarily alter the typical DMN-ECN anti-correlation, allowing for more flexible, context-dependent coordination between these networks. This altered connectivity pattern may facilitate integration of emotional experiences that are typically dissociated or compartmentalized. For example, hypnotic suggestions for accessing and processing traumatic memories may be effective precisely because the altered network configuration allows for simultaneous engagement of emotional memory systems (DMN) and regulatory control systems (ECN), enabling processing and integration rather than re-traumatization or avoidance.

With repeated hypnotic practice, individuals may develop more flexible DMN-ECN connectivity patterns that persist beyond hypnotic states, supporting greater emotional coherence and reduced dissociative fragmentation in daily life. This represents a form of neuroplastic change in which repeated experiences of integrated network functioning gradually alter baseline network organization.

#### 5.2.4. Pathway 4: Reduced Defensive Processing → Compassionate Self-Awareness

The relaxed, absorbed quality of hypnotic states may reduce activation of defensive psychological processes that typically interfere with compassionate self-awareness. Defensive processes such as denial, rationalization, and self-enhancement serve to protect self-esteem and reduce anxiety, but they can also prevent genuine self-awareness and self-acceptance (Cramer, 2006).

During hypnosis, the combination of reduced DMN activity (less rigid self-concept activation), enhanced ECN-SaN coupling (focused yet flexible attention), and the safe, supportive therapeutic context may create conditions in which defensive processes are less necessary. This allows for more direct, honest self-observation without the usual layers of defensive distortion. Hypnotic suggestions for self-compassion, self-acceptance, or self-forgiveness may be particularly effective in this context, as they are less likely to be filtered through defensive processes.

Additionally, the dissociative quality of hypnotic experience—the sense of observing oneself from a slight distance—may facilitate compassionate self-awareness by creating psychological space between the observing self and the observed self. This “decentered” perspective allows for observation of one’s own thoughts, emotions, and behaviors with less identification and reactivity, similar to the decentering cultivated in mindfulness meditation (Fresco et al., 2007). From this decentered perspective, self-compassion becomes more accessible, as one can observe personal struggles and suffering with the same kindness one might extend to a friend.

### 5.3. Integration and Emergence of FP

The four pathways described above operate in parallel and interact synergistically to produce the integrated state of Fundamental Peace. FP is not simply the sum of its components but represents an emergent state in which the components mutually reinforce one another:

- Reduced self-referential rigidity (Pathway 1) facilitates flexible attentional control (Pathway 2) by reducing the pull of habitual self-focused thoughts.
- Flexible attentional control (Pathway 2) supports emotional coherence (Pathway 3) by enabling sustained attention to emotional experiences without avoidance or overwhelm.
- Emotional coherence (Pathway 3) enhances compassionate self-awareness (Pathway 4) by providing access to a fuller range of self-experiences.
- Compassionate self-awareness (Pathway 4) further reduces self-referential rigidity (Pathway 1) by decreasing defensive self-concept maintenance.

This positive feedback loop suggests that once FP begins to emerge, it may become self-sustaining, at least temporarily. With repeated practice, the neural and cognitive patterns associated with FP may become more readily accessible and more stable, requiring less external support (hypnotic induction) to maintain. This is consistent with clinical observations that individuals who practice self-hypnosis regularly often report increasing ease in accessing peaceful, integrated states and increasing carryover of these states into daily life.

### 5.4. Individual Differences and Moderating Factors

The pathways from hypnotic induction to FP emergence are moderated by several individual difference factors:

**Hypnotic suggestibility** is the most obvious moderator, with highly suggestible individuals typically showing more pronounced network changes and stronger subjective responses to hypnotic suggestions (Hoeft et al., 2012). However, even individuals with moderate suggestibility may experience meaningful FP states, particularly with extended practice and individualized approaches.

**Baseline network organization** may also moderate responses. Individuals with hyperactive DMN at baseline (e.g., those with depression or anxiety characterized by rumination) may show particularly pronounced benefits from DMN reduction during hypnosis. Conversely, individuals with already well-regulated network dynamics may show smaller changes but may still benefit from the enhanced integration and flexibility that hypnosis facilitates.

**Trauma history and dissociative tendencies** represent complex moderating factors. On the one hand, individuals with trauma histories may have greater need for the integration and emotional coherence that FP provides. On the other hand, dissociative defenses may interfere with the integration processes, and hypnotic procedures must be carefully adapted to avoid triggering overwhelming emotional activation or further dissociation (van der Hart et al., 2006).

**Therapeutic relationship and context** also moderate outcomes. The safety, trust, and rapport established between clinician and client create the interpersonal context in which hypnotic work occurs. A strong therapeutic alliance may enhance willingness to engage with difficult emotional material and may provide the secure base necessary for reorganizing self-experience (H. Spiegel & Spiegel, 2004).

## 6. Evidence Evaluation and Alternative Theoretical Frameworks

### 6.1. Quality of Evidence for Network Changes

The evidence for hypnotic modulation of large-scale brain networks comes primarily from neuroimaging studies using fMRI, PET, and EEG. While this literature has grown substantially in recent decades, several limitations must be acknowledged.

First, sample sizes in neuroimaging studies of hypnosis have typically been small, often including fewer than 30 participants. Recent work on reproducibility in neuroimaging has demonstrated that brain-wide association studies require samples of hundreds or thousands of participants to achieve adequate statistical power and reproducibility (Marek et al., 2022). Most existing hypnosis neuroimaging studies fall far short of these standards, raising concerns about the reliability and generalizability of reported findings.

Second, there is considerable heterogeneity across studies in hypnotic induction procedures, suggestion content, control conditions, and analysis methods. This heterogeneity makes it difficult to synthesize findings across studies and to determine which network changes are consistent and replicable versus which may be artifacts of specific methodological choices.

Third, most studies have focused on highly hypnotizable individuals, with limited examination of network changes in low or moderate hypnotizable individuals. This sampling bias limits understanding of the full range of neural responses to hypnosis and may inflate effect size estimates.

Fourth, the causal relationships between network changes and subjective experiences remain unclear. While correlational studies have identified associations between network activity patterns and hypnotic depth or responsiveness to suggestions, experimental studies that manipulate network activity to examine effects on hypnotic experiences are lacking. Emerging techniques such as transcranial magnetic stimulation (TMS) or transcranial direct current stimulation (tDCS) could potentially be used to test causal hypotheses, but such studies have not yet been conducted.

Despite these limitations, the consistency of certain findings across multiple studies—particularly DMN reduction during hypnotic induction—provides reasonable confidence that hypnosis does produce measurable changes in large-scale brain network activity. However, the specific details of these changes and their functional significance require further investigation with larger samples, standardized protocols, and more sophisticated analysis methods.

### 6.2. Alternative Theoretical Frameworks

Several alternative theoretical frameworks have been proposed to explain hypnotic phenomena, each emphasizing different mechanisms and processes. Our integrative model must be evaluated in relation to these alternatives.

Dissociated Control Theory (Woody & Bowers, 1994) proposes that hypnotic responses result from a dissociation between executive control systems and lower-level cognitive processes. According to this theory, hypnotic suggestions create a situation in which executive control systems initiate responses but then become dissociated from monitoring and control of those responses, producing the subjective experience of involuntariness that characterizes hypnotic phenomena. This theory has been influential and is supported by evidence that hypnotic responses often feel automatic or involuntary despite being goal-directed.

Our model is compatible with dissociated control theory but extends it by specifying the neural mechanisms (altered DMN-ECN connectivity) that may underlie dissociation between executive control and monitoring systems. Additionally, our model emphasizes that hypnosis can facilitate the integration of dissociated self-states rather than only producing dissociation. The key distinction is between adaptive, temporary dissociation (as in hypnotic responses) and maladaptive, chronic dissociation (as in dissociative disorders).

Cold Control Theory (Dienes & Perner, 2007) proposes that hypnotic responses involve higher-order thoughts about mental states rather than changes in the mental states themselves. According to this theory, hypnotic suggestions alter metacognitive awareness—the awareness of one’s own mental states—rather than the mental states directly. For example, a suggestion for arm levitation might not directly cause the arm to rise but might cause the person to be unaware of their intention to raise the arm, producing the subjective experience of involuntary movement.

Our model acknowledges the importance of metacognitive processes but proposes that hypnosis involves changes in both first-order mental states (through network modulation) and higher-order awareness of those states. The network changes we describe (DMN reduction, ECN-SaN coupling) represent alterations in the neural systems that generate first-order experiences, not merely changes in awareness of those experiences. However, metacognitive changes are likely also involved, particularly in producing the subjective quality of involuntariness and absorption.

Predictive Processing Accounts (Oakley & Halligan, 2013) frame hypnotic phenomena in terms of predictive coding and active inference. According to these accounts, hypnotic suggestions function as strong prior expectations that shape perception and experience through top-down predictive signals. When suggestions are sufficiently strong and precise, they can override bottom-up sensory evidence, producing alterations in perception, cognition, or behavior that align with the suggested expectation.

Our model is highly compatible with predictive processing accounts and can be viewed as specifying the neural network mechanisms through which hypnotic suggestions exert their top-down effects. The enhanced ECN-SaN coupling we describe may reflect strengthened top-down predictive signals, while DMN reduction may reflect decreased interference from self-referential predictions. Future work integrating our network-based model with computational predictive processing models could provide a more complete mechanistic account.

Social-Cognitive Models (Lynn et al., 2015) emphasize the role of social context, expectations, attitudes, and motivations in shaping hypnotic responses. According to these models, hypnotic phenomena result from the interaction of multiple factors including hypnotic suggestibility (a trait), situational factors (context, rapport, expectations), and cognitive processes (imagination, attention, interpretation). Social-cognitive models tend to be skeptical of “special state” theories that posit hypnosis as a unique altered state of consciousness.

Our model does not require a “special state” interpretation but does propose that hypnotic procedures produce measurable changes in brain network organization that differ from ordinary waking consciousness. These network changes are not inconsistent with social-cognitive factors; rather, social-cognitive factors (expectations, rapport, motivation) likely influence the degree and nature of network changes that occur. An integrative account would recognize that both neural mechanisms and social-cognitive factors contribute to hypnotic phenomena, with neither level of explanation being reducible to the other.

**Figure 2:**
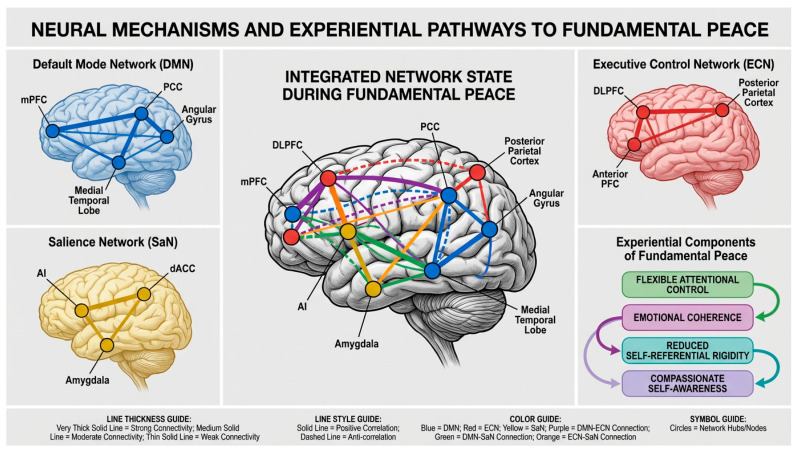
**Neural Mechanisms and Experiential Pathways to Fundamental Peace. Overall structure:** Four-quadrant layout showing: (**Top left**): DMN regions and connectivity; (**Top right**): ECN regions and connectivity; (**Bottom left**): SaN regions and connectivity; (**Center (improved)**): Integrated network configuration during FP state; (**Bottom right**): Experiential components of FP.

See Figure 2 for the overall structure of the Neural Mechanisms and Experimental Pathways to Fundamental Peace

### 6.3. Testable Predictions

A key strength of our mechanistic model is that it generates specific, testable predictions that can be evaluated in future research:

**Prediction 1:** Individuals who show greater DMN reduction during hypnotic induction will report greater reductions in self-referential rigidity and rumination, both during hypnosis and in daily life following repeated hypnotic practice.

**Prediction 2:** Interventions that enhance ECN-SaN coupling (through neurofeedback, brain stimulation, or targeted cognitive training) will enhance hypnotic responsiveness and facilitate access to FP states.

**Prediction 3:** Individuals with trauma histories and dissociative symptoms will show altered DMN-ECN connectivity patterns at baseline, and hypnotic interventions that normalize these connectivity patterns will produce improvements in emotional coherence and reductions in dissociative symptoms.

**Prediction 4:** The four components of FP (flexible attentional control, emotional coherence, reduced self-referential rigidity, compassionate self-awareness) will show positive correlations with one another and will collectively predict better emotion regulation outcomes than any single component alone.

**Prediction 5:** Longitudinal practice of self-hypnosis will produce lasting changes in resting-state network organization, particularly increased flexibility in DMN-ECN connectivity, and these network changes will mediate improvements in emotion regulation and well-being.

**Prediction 6:** The relationship between hypnotic suggestibility and clinical outcomes will be mediated by the degree of network reconfiguration achieved during hypnotic sessions, such that individuals who show pronounced network changes will benefit more from hypnotic interventions regardless of their baseline suggestibility scores.

These predictions can be tested using combinations of neuroimaging, behavioral assessment, and clinical outcome measures in both cross-sectional and longitudinal designs. Rigorous testing of these predictions will be essential for validating, refining, or potentially falsifying the proposed model.

## 7. Clinical Applications and Implementation

### 7.1. Trauma Treatment and Dissociation

One of the most promising applications of our integrative framework is in the treatment of trauma-related disorders, particularly those involving dissociative symptoms. Traditional trauma treatments such as prolonged exposure therapy or cognitive processing therapy focus on processing traumatic memories through repeated exposure and cognitive restructuring (Foa et al., 2007). While these approaches are effective for many individuals, they can be challenging for those with significant dissociative symptoms, who may have difficulty accessing traumatic memories in a coherent way or may become overwhelmed by emotional activation during exposure.

Hypnotic approaches to trauma treatment offer several potential advantages. First, the altered network configuration during hypnosis—particularly the modified DMN-ECN connectivity—may allow for accessing traumatic memories while maintaining sufficient regulatory control to prevent overwhelming emotional activation. This creates a “window of tolerance” in which traumatic material can be processed without triggering dissociative defenses or emotional flooding (van der Hart et al., 2006).

Second, hypnotic techniques can be used to work directly with dissociated self-states or ego states, facilitating communication and integration between parts of the self that are typically compartmentalized (Watkins & Watkins, 1997). For example, hypnotic suggestions can help establish internal communication between a traumatized child ego state and an adult ego state, allowing for the adult self to provide comfort and protection to the child self. Over time, this internal communication can facilitate integration of dissociated experiences into a more coherent autobiographical narrative.

Third, hypnotic interventions can help establish internal resources and safe places that trauma survivors can access when working with difficult material. Suggestions for creating an internal “safe place” or for accessing inner strength and resilience can provide the psychological scaffolding necessary for trauma processing. These resources can be anchored through post-hypnotic suggestions, making them accessible outside of formal hypnotic sessions.

Clinical protocols for hypnotic trauma treatment typically follow a phase-based approach (van der Hart et al., 2006): Phase 1 focuses on stabilization, resource building, and establishing safety; Phase 2 involves processing traumatic memories and integrating dissociated self-states; and Phase 3 emphasizes integration, rehabilitation, and reconnection with life. Hypnotic techniques can be valuable in all three phases but are particularly useful in Phases 1 and 2.

It is crucial to note that hypnotic trauma treatment requires specialized training and should only be conducted by clinicians with expertise in both hypnosis and trauma treatment. Poorly conducted hypnotic work with trauma survivors can potentially exacerbate dissociation or trigger overwhelming emotional activation. Careful assessment, pacing, and attention to the therapeutic relationship are essential.

### 7.2. Emotion Regulation Training

Beyond trauma treatment, hypnotic approaches can be used to enhance general emotion regulation capacities in individuals with anxiety, depression, or emotion dysregulation. The network changes produced by hypnosis—particularly enhanced ECN-SaN coupling and reduced DMN rigidity—directly target neural systems involved in emotion regulation.

Hypnotic emotion regulation training might include:

- **Suggestions for emotional flexibility:** Teaching individuals to shift between emotional states adaptively, rather than becoming stuck in particular emotional patterns. For example, suggestions might facilitate moving from anxiety to calm, or from sadness to acceptance, while maintaining awareness of the full range of emotional experience.
- **Reappraisal training under hypnosis:** Using the enhanced suggestibility of hypnotic states to practice cognitive reappraisal of emotional situations. The reduced defensive processing during hypnosis may allow for more genuine reappraisal rather than superficial rationalization.
- **Somatic regulation techniques:** Using hypnotic suggestions to modulate physiological arousal, heart rate, breathing, and muscle tension. These somatic changes can then serve as anchors for emotional regulation in daily life.
- **Compassionate self-talk:** Establishing internal dialogues characterized by self-compassion and self-acceptance, which can be anchored through post-hypnotic suggestions and practiced in daily life.

Research on hypnotic emotion regulation training is still limited, but preliminary evidence suggests that such approaches can produce improvements in emotion regulation capacity that persist beyond the hypnotic sessions themselves (Jensen et al., 2015). Combining hypnotic training with other evidence-based emotion regulation interventions (such as dialectical behavior therapy skills training) may produce synergistic effects.

### 7.3. Integration with Other Therapeutic Approaches

Hypnotic interventions are rarely used in isolation but are typically integrated with other therapeutic approaches. Our mechanistic framework suggests several natural points of integration:

**Cognitive-Behavioral Therapy (CBT):** Hypnotic techniques can enhance CBT by facilitating cognitive restructuring, exposure exercises, and behavioral activation. The reduced defensive processing during hypnosis may allow for more genuine examination of maladaptive thoughts, while the enhanced suggestibility may strengthen the impact of cognitive interventions.

**Mindfulness-Based Interventions:** Hypnosis and mindfulness share several features, including focused attention, present-moment awareness, and non-judgmental observation (Raz & Lifshitz, 2016). Integrating hypnotic and mindfulness practices may produce complementary benefits, with hypnosis providing more directive, goal-oriented interventions and mindfulness providing more open, receptive practices.

**Psychodynamic Therapy:** Hypnotic techniques have long been used in psychodynamic contexts to access unconscious material, work with resistance, and facilitate insight (Fromm & Nash, 1992). Our network-based model provides a contemporary neuroscience framework for understanding how hypnosis might facilitate access to material that is typically outside of conscious awareness.

**Somatic Therapies:** Approaches such as Somatic Experiencing (Levine, 2010) and Sensorimotor Psychotherapy (Ogden et al., 2006) emphasize working with bodily sensations and physiological arousal in trauma treatment. Hypnotic techniques for modulating somatic experience can be readily integrated with these approaches.

### 7.4. Training and Competency Requirements

The effective and ethical use of clinical hypnosis requires appropriate training and ongoing supervision. While formal certification is not legally required for licensed mental health professionals to use hypnosis in most jurisdictions, professional organizations strongly recommend specialized training (Lynn et al., 2015). It is important to clarify that formal hypnosis training is recommended for optimal clinical practice but is not an absolute requirement for licensed psychiatrists, psychologists, and other mental health professionals who wish to incorporate hypnotic techniques into their practice.

Recommended training pathways include:

- **Workshops and certification programs** offered by professional organizations such as the American Society of Clinical Hypnosis (ASCH), the Society for Clinical and Experimental Hypnosis (SCEH), or the British Society of Clinical and Academic Hypnosis (BSCAH). These programs typically include 40–60 h of initial training covering theory, assessment, induction techniques, and clinical applications, followed by advanced training in specialized applications.
- **Supervised clinical practice** with experienced hypnosis practitioners, allowing for feedback and refinement of skills in real clinical contexts.
- **Ongoing education** through conferences, advanced workshops, and consultation groups to stay current with evolving research and practice standards.

Competency in clinical hypnosis includes not only technical skills (induction techniques, suggestion formulation) but also:

- Understanding of hypnotic phenomena and individual differences in responsiveness;
- Ability to assess appropriateness of hypnotic interventions for specific clients and presenting problems;
- Knowledge of contraindications and potential adverse effects;
- Ethical awareness regarding issues such as false memories, dependency, and boundary management;
- Integration of hypnotic techniques within a broader therapeutic framework.

For trauma treatment applications specifically, additional specialized training in trauma-informed care and dissociation is essential. Clinicians should not attempt hypnotic trauma work without adequate preparation and supervision, given the potential for iatrogenic harm if hypnotic techniques are applied inappropriately with trauma survivors.

## 8. Discussion and Future Directions

### 8.1. Theoretical Contributions and Limitations

This integrative review has proposed a comprehensive mechanistic framework linking hypnotic modulation of large-scale brain networks to emotion regulation and self-integration, culminating in the emergence of Fundamental Peace. The framework makes several theoretical contributions:

First, it provides an explicit multi-level model connecting neural mechanisms (network dynamics), cognitive processes (attention, emotion regulation, self-referential processing), and experiential outcomes (FP components). This multi-level integration addresses a significant gap in the hypnosis literature, which has often focused on single levels of analysis without adequately connecting across levels.

Second, it introduces the construct of Fundamental Peace as a measurable neuro-experiential state that can serve as a target for both research and clinical intervention. By operationalizing FP in terms of specific components with distinct neural correlates, the framework provides a foundation for rigorous empirical investigation.

Third, it integrates contemporary neuroscience perspectives on large-scale brain networks with clinical hypnosis theory and practice, providing a bridge between basic neuroscience research and clinical application. This integration may facilitate translation of neuroscience findings into improved clinical interventions.

Fourth, it generates specific, testable predictions that can guide future research and that could potentially falsify the model if not supported by empirical evidence.

However, several important limitations must be acknowledged:

The evidence base for specific components of the model remains incomplete. While DMN reduction during hypnosis is relatively well-established, the evidence for specific patterns of ECN-SaN coupling and DMN-ECN connectivity changes is more limited and inconsistent across studies. The causal relationships between network changes and experiential outcomes remain largely inferential rather than empirically demonstrated.

**The construct of Fundamental Peace, while conceptually distinct from related constructs, requires empirical validation.** No validated measurement instrument currently exists, and the proposed four-component structure requires psychometric evaluation. It remains to be determined whether FP represents a coherent construct or whether the four components are better understood as separate dimensions.

**The model has been developed primarily based on research with Western, educated, industrialized, rich, and democratic (WEIRD) populations.** The extent to which the proposed mechanisms and outcomes generalize across cultural contexts remains unclear. Cultural factors may influence both the phenomenology of hypnotic experiences and the value placed on constructs such as self-integration and emotional coherence.

**The clinical applications described are based on theoretical reasoning and limited clinical evidence rather than on large-scale randomized controlled trials.** While case studies and small clinical trials support the potential utility of hypnotic approaches for trauma and emotion regulation, more rigorous outcome research is needed to establish efficacy and to identify for whom and under what conditions hypnotic interventions are most beneficial.

### 8.2. Linking Mechanistic Model to FP Emergence

A critical aspect of our framework that requires explicit articulation is how the mechanistic model’s predicted network dynamics directly relate to the emergence and measurement of Fundamental Peace. This linkage represents the core theoretical contribution of our integrative model and warrants detailed explication.

The emergence of FP is not simply a correlate of network changes but is proposed to be a direct consequence of specific network configurations:

**DMN Reduction → Reduced Self-Referential Rigidity (FP Component 3):** The reduction in posterior cingulate cortex and medial prefrontal cortex activity during hypnosis directly produces decreased activation of rigid self-concepts and reduced engagement in repetitive self-referential thinking. This neural change is experienced subjectively as greater flexibility in self-representation and reduced rumination. Measurement of this component can be achieved through both neural markers (resting-state DMN connectivity) and behavioral/self-report measures (rumination scales, self-concept flexibility tasks).

**ECN-SaN Coupling → Flexible Attentional Control (FP Component 1):** Enhanced functional connectivity between the dorsolateral prefrontal cortex (ECN) and anterior cingulate/insula (SaN) produces coordinated attentional control in which salience detection and goal-directed focus operate synergistically rather than competitively. This neural configuration is experienced as effortless attention—focused yet flexible, sustained yet not rigid. Measurement can include task-based fMRI during attention tasks, behavioral measures of sustained attention and attentional flexibility, and self-report measures of absorption and flow.

**Altered DMN-ECN Connectivity → Emotional Coherence (FP Component 2):** The shift from rigid anti-correlation to more flexible, context-dependent coordination between the DMN and ECN enables simultaneous access to emotional memories (DMN-mediated) and regulatory control (ECN-mediated). This neural integration is experienced as emotional coherence—the ability to access and integrate emotional experiences across self-states without dissociative fragmentation. Measurement can include resting-state functional connectivity analysis, narrative coherence coding of autobiographical memories, and clinical assessment of dissociative symptoms.

**Reduced Defensive Processing → Compassionate Self-Awareness (FP Component 4):** The combined effects of DMN reduction, ECN-SaN coupling, and altered DMN-ECN connectivity create a neural state in which defensive processing is less necessary and less active. This is experienced as the capacity for compassionate self-observation—the ability to witness one’s own experiences with kindness and acceptance. Measurement can include self-report measures of self-compassion, behavioral measures of self-critical versus self-compassionate responses to failure, and neural measures of self-referential processing during self-evaluation tasks.

Critically, these four pathways are not independent but interact synergistically to produce an integrated FP state. The emergence of FP represents a phase transition in which the components mutually reinforce one another, creating a stable (though temporary) configuration. This emergent quality means that FP is more than the sum of its components—it represents a qualitatively distinct experiential state with its own phenomenological characteristics.

Measurement of FP emergence should therefore employ multi-method approaches that assess:

1. **Neural markers:** Specific network configurations (DMN activity, ECN-SaN coupling, DMN-ECN connectivity) measured via fMRI or EEG.
2. **Cognitive-behavioral markers:** Performance on tasks assessing attentional flexibility, emotion regulation, self-referential processing, and narrative coherence.
3. **Experiential markers:** Self-report measures capturing the subjective qualities of FP components.
4. **Temporal dynamics:** Assessment of how quickly FP states emerge, how long they persist, and how readily they can be re-accessed.

This explicit linkage between neural mechanisms and experiential outcomes provides the foundation for rigorous empirical testing of the model and for developing targeted interventions that optimize network configurations to facilitate FP emergence.

### 8.3. Cultural and Ethical Considerations

The constructs and mechanisms described in this review reflect, to a significant degree, Western individualistic values and assumptions about the self, emotion, and well-being. Several aspects of the framework warrant critical examination from cross-cultural perspectives.

The emphasis on self-integration and coherent self-narrative reflects Western psychological assumptions about the self as a unified, continuous entity. In contrast, many non-Western cultural traditions conceptualize the self as fundamentally relational, contextual, or even illusory (Markus & Kitayama, 1991). The value placed on emotional coherence and reduced self-referential rigidity may not be universally shared. Some cultural contexts may value emotional restraint, social harmony, or acceptance of self-contradiction over the kind of integrated self-awareness emphasized in our FP construct.

The construct of Fundamental Peace itself, while drawing on contemplative traditions that originated in Eastern cultures, has been operationalized here in ways that may reflect Western psychological frameworks. The emphasis on individual emotional regulation capacity, personal self-awareness, and autonomous attentional control reflects individualistic values. Collectivist cultural contexts might emphasize relational harmony, social role fulfillment, or spiritual transcendence over individual psychological integration.

Hypnotic practices and their meanings vary considerably across cultural contexts. While hypnosis as a formal clinical technique is primarily a Western phenomenon, trance states and altered consciousness practices exist in virtually all cultures, often embedded in religious, healing, or community rituals (Krippner & Sulla, 2000). The mechanisms and outcomes of these culturally embedded practices may differ from those of clinical hypnosis as practiced in Western medical and psychological settings.

These cultural considerations have important implications for research and practice:

- Research on hypnosis and FP should include diverse cultural populations and should examine whether the proposed mechanisms and outcomes generalize across cultural contexts;
- Measurement instruments for FP and related constructs should be validated cross-culturally and may need to be adapted to reflect culturally specific values and experiences;
- Clinical applications of hypnosis should be culturally adapted, taking into account clients’ cultural backgrounds, values, and beliefs about the self, emotion, and healing;
- Researchers and clinicians should maintain critical awareness of the cultural assumptions embedded in their theoretical frameworks and should remain open to alternative conceptualizations.

Ethical considerations in hypnosis research and practice include:

**Informed consent:** Clients should be fully informed about the nature of hypnotic procedures, potential benefits and risks, and alternatives to hypnotic treatment. The common misconception that hypnosis involves loss of control or that the hypnotist can make people do things against their will should be explicitly addressed.

**False memories:** Hypnotic procedures, particularly those involving age regression or memory recovery, carry risk of creating false memories or distorting existing memories (Lynn et al., 2015). Clinicians should avoid suggestive questioning about past events and should be aware that memories retrieved under hypnosis may not be historically accurate, even if they feel vivid and real to the client.

**Dependency:** Some clients may develop excessive reliance on hypnotic interventions or on the hypnotist, potentially interfering with development of autonomous self-regulation capacities. Clinicians should emphasize self-hypnosis training and should work toward client independence rather than ongoing dependency.

**Boundary issues:** The intimate, focused nature of hypnotic work requires careful attention to therapeutic boundaries. The power differential inherent in the hypnotic relationship should be acknowledged and managed ethically.

**Scope of practice:** Clinicians should only use hypnotic techniques within their areas of competence and should refer clients to specialists when appropriate. Hypnosis should not be used as a substitute for necessary medical or psychiatric treatment.

### 8.4. Future Research Directions

Several research directions emerge as priorities for advancing understanding of hypnosis, emotion regulation, and Fundamental Peace:

#### 8.4.1. Large-Scale Neuroimaging Studies

Future research should employ larger samples (hundreds to thousands of participants) to achieve adequate statistical power and reproducibility. Multi-site collaborations using standardized protocols could help address the sample size limitations of existing studies. Advanced network analysis methods, including dynamic functional connectivity analysis and graph-theoretical approaches, could provide more nuanced understanding of network reconfiguration during hypnosis.

#### 8.4.2. Causal Inference Methods

Moving beyond correlational neuroimaging to experimental manipulation of network activity could test causal hypotheses. Techniques such as transcranial magnetic stimulation (TMS), transcranial direct current stimulation (tDCS), or neurofeedback could be used to modulate specific network configurations and examine effects on hypnotic responsiveness and FP emergence. Randomized controlled trials comparing hypnotic interventions to active control conditions could establish causal relationships between hypnotic procedures and clinical outcomes.

#### 8.4.3. Longitudinal Studies

Tracking changes in network organization, emotion regulation capacity, and FP over extended periods of hypnotic practice could reveal neuroplastic changes and identify mechanisms of lasting therapeutic benefit. Longitudinal designs could also examine individual trajectories of change, identifying for whom hypnotic interventions are most beneficial and what factors predict positive outcomes.

#### 8.4.4. Mechanism-Focused Clinical Trials

Clinical trials that explicitly test mechanistic hypotheses—for example, examining whether network changes mediate clinical outcomes—could validate or refine the proposed model. Trials could also compare different hypnotic approaches (e.g., Ericksonian vs. traditional, direct vs. indirect suggestions) to determine which procedures most effectively produce desired network configurations and clinical outcomes.

#### 8.4.5. Development and Validation of FP Measures

Creating and validating measurement instruments for Fundamental Peace and its components is essential for rigorous empirical investigation. This work should include psychometric evaluation (reliability, validity, factor structure), examination of neural correlates, and assessment of sensitivity to change following interventions.

#### 8.4.6. Cross-Cultural Research

Examining hypnotic phenomena, network dynamics, and FP across diverse cultural contexts could reveal universal versus culturally specific aspects of the proposed mechanisms. Such research could also identify culturally adapted approaches to hypnotic intervention that are more effective for specific populations.

#### 8.4.7. Integration with Computational Modeling

Developing computational models that simulate network dynamics during hypnosis could provide more precise mechanistic accounts and could generate novel predictions. Predictive processing models, in particular, could be integrated with network-based models to provide a more complete account of how hypnotic suggestions alter perception and experience.

#### 8.4.8. Comparative Studies with Other Interventions

Comparing hypnotic approaches to other interventions that target similar mechanisms (e.g., meditation, neurofeedback, psychedelic-assisted therapy) could reveal common and distinct pathways to emotion regulation and self-integration. Such comparative research could inform development of integrated or sequential treatment protocols that optimize outcomes.

## 9. Conclusions

This integrative review has proposed a comprehensive mechanistic framework for understanding how hypnotic phenomena modulate large-scale brain networks to facilitate emotion regulation and self-integration, culminating in the emergence of Fundamental Peace. By synthesizing evidence from cognitive neuroscience, affective science, and clinical hypnosis research, we have articulated explicit pathways linking neural mechanisms to psychological processes and experiential outcomes.

The framework makes several key contributions. First, it provides a multi-level integrative model that connects neural network dynamics (DMN reduction, ECN-SaN coupling, altered DMN-ECN connectivity) to specific cognitive and emotional processes (attentional control, emotion regulation, self-referential processing) and measurable experiential outcomes (the four components of Fundamental Peace). Second, it introduces Fundamental Peace as a novel construct that captures an integrated state of emotional and self-regulatory capacity, distinct from related constructs such as equanimity, well-being, or mindfulness. Third, it generates specific, testable predictions that can guide future research and that provide opportunities for empirical validation or falsification of the model.

The clinical implications of this framework are substantial. For trauma treatment, the model suggests that hypnotic interventions can facilitate integration of dissociated self-states by temporarily altering the typical anti-correlation between the DMN and ECN, creating a neural configuration that allows for processing traumatic memories while maintaining regulatory control. For emotion regulation training more broadly, hypnotic approaches can target the specific network configurations that support flexible attentional control, emotional coherence, and compassionate self-awareness. Integration of hypnotic techniques with other evidence-based therapeutic approaches may produce synergistic effects, enhancing outcomes across diverse clinical presentations.

Important limitations must be acknowledged. The evidence base for specific components of the model remains incomplete, with many proposed mechanisms requiring further empirical validation. The construct of Fundamental Peace requires psychometric development and validation. Cultural generalizability of the framework is uncertain, and ethical considerations regarding false memories, dependency, and scope of practice must be carefully managed in clinical applications.

Future research employing large-scale neuroimaging studies, causal inference methods, longitudinal designs, and cross-cultural approaches will be essential for validating, refining, or potentially revising the proposed framework. Development of validated measurement instruments for FP and its components will enable rigorous empirical investigation. Mechanism-focused clinical trials can test whether the proposed pathways actually mediate clinical outcomes and can identify optimal approaches for facilitating FP emergence.

Ultimately, this integrative framework aims to advance both the scientific understanding of hypnotic phenomena and clinical application of hypnotic interventions. By providing explicit mechanistic accounts linking neural, cognitive, and experiential levels of analysis, the framework offers a foundation for developing more effective, targeted interventions for emotion regulation difficulties, trauma-related disorders, and the cultivation of integrated psychological well-being. As research continues to elucidate the mechanisms through which hypnosis modulates consciousness, emotion, and self-experience, the potential for hypnotic approaches to contribute to both treatment of psychopathology and enhancement of human flourishing becomes increasingly apparent.

## Figures and Tables

**Table 1:** Components of Fundamental Peace and Their Neural Correlates.

FP Component	Operational Definition	Neural Correlates	Measurement Approaches
Flexible Attentional Control	Capacity to direct and sustain attention without effortful suppression	Enhanced ECN-SaN coupling; Increased dlPFC-ACC connectivity	Sustained attention tasks; Attentional flexibility measures; Self-report absorption scales
Emotional Coherence	Integration across self-states without dissociative fragmentation	Flexible DMN-ECN connectivity; Reduced DMN-ECN anti-correlation	Narrative coherence coding; Dissociative symptoms scales; Autobiographical memory integration tasks
Reduced Self-Referential Rigidity	Decreased rigid, repetitive self-focused thinking	Reduced DMN activity (PCC, mPFC); Decreased DMN connectivity	Rumination scales; Self-concept flexibility tasks; Mind-wandering measures
Compassionate Self-Awareness	Non-judgmental observation of own experiences with kindness	Reduced defensive processing; Balanced DMN-ECN coordination	Self-compassion scales; Self-critical response measures; Decentering questionnaires

**Legend: FP** = Fundamental Peace; **ECN** = Executive Control Network; **SaN** = Salience Network; **DMN** = Default Mode Network; **dlPFC** = Dorsolateral Prefrontal Cortex; **ACC** = Anterior Cingulate Cortex; **PCC** = Posterior Cingulate Cortex; **mPFC** = Medial Prefrontal Cortex. **Note:** The distinction between the two instances of “FP” in the original table has been clarified—both refer to Fundamental Peace as the overarching construct, with the table rows describing its four constituent components.

**Table 2:** Fundamental Peace vs. Related Constructs.

Construct	Definition	Key Features	Neural Correlates	Measurement	Distinction from Fundamental Peace
Fundamental Peace	Dynamic integrated emotion regulation capacity	Flexible attention, emotional coherence, reduced self-rigidity, compassionate awareness	Balanced DMN-ECN-SaN coordination; flexible network reconfiguration	FP Scale (proposed); network flexibility metrics; behavioral regulation tasks	Emphasizes dynamic regulatory capacity, not static state
Equanimity (Desbordes et al., 2015)	Even-minded mental state; affective neutrality	Non-reactivity to pleasant/unpleasant; balanced affect	Reduced amygdala reactivity; altered insula-amygdala connectivity	Equanimity Scale; affect ratings	FP includes active engagement, not just neutrality; emphasizes coherence across self-states
Psychological Well-Being (Ryff, 2014)	Positive evaluation of life and self	Autonomy, mastery, growth, purpose, positive relations, self-acceptance	Diverse; includes reward circuits, prefrontal regions	Ryff Scales of Psychological Well-Being	FP is regulatory capacity, not evaluative judgment; can exist amid challenges
Nondual Awareness (Josipovic, 2014)	Awareness without subject-object division	Dissolution of self-other boundary; pure consciousness	Reduced DMN activity; altered posterior cingulate function	Nondual Awareness Dimensional Assessment	FP maintains functional self-awareness; emphasizes integration, not dissolution
Flow (Csikszentmihalyi, 1990)	Optimal experience during challenging activity	Absorption, loss of self-consciousness, time distortion	Transient hypofrontality; reduced DMN activity	Flow State Scale	FP is stable capacity, not task-specific state; includes self-awareness
Mindfulness (Kabat-Zinn, 1990)	Present-moment awareness with acceptance	Non-judgmental attention to present	Altered DMN-ECN connectivity; increased insula activation	FFMQ, MAAS	FP emphasizes emotional coherence and self-integration more than present-focus

## Data Availability

The original contributions presented in this study are included in the article. Further inquiries can be directed to the corresponding author.

## Abbreviations

The following abbreviations are used in this manuscript:

DMN	Default Mode Network
ECN	Executive Control Network
SaN	Salience Network
FP	Fundamental Peace
HIP	Hypnotic Induction Profile
fMRI	Functional Magnetic Resonance Imaging
ACC	Anterior Cingulate Cortex
PFC	Prefrontal Cortex
PTSD	Post-Traumatic Stress Disorder
